# Association between sex and survival after out‐of‐hospital cardiac arrest: A systematic review and meta‐analysis

**DOI:** 10.1002/emp2.12943

**Published:** 2023-04-29

**Authors:** Laura A. E. Bijman, Raied Alotaibi, Caroline A. Jackson, Gareth Clegg, Nynke Halbesma

**Affiliations:** ^1^ Usher Institute University of Edinburgh Edinburgh UK; ^2^ Resuscitation Research Group The University of Edinburgh Edinburgh UK

**Keywords:** heart arrest, out‐of‐hospital cardiac arrest, sex, survival, systematic review

## Abstract

The current literature on sex differences in 30‐day survival following out‐of‐hospital cardiac arrest (OHCA) is conflicting, with 3 recent systematic reviews reporting opposing results. To address these contradictions, this systematic literature review and meta‐analysis aimed to synthesize the literature on sex differences in survival after OHCA by including only population‐based studies and through separate meta‐analyses of crude and adjusted effect estimates. MEDLINE and Embase databases were systematically searched from inception to March 23, 2022 to identify observational studies reporting sex‐specific 30‐day survival or survival until hospital discharge after OHCA. Two meta‐analyses were conducted. The first included unadjusted effect estimates of the association between sex and survival (comparing males vs females), whereas the second included effect estimates adjusted for possible mediating and/or confounding variables. The PROSPERO registration number was CRD42021237887, and the search identified 6712 articles. After the screening, 164 potentially relevant articles were identified, of which 26 were included. The pooled estimate for crude effect estimates (odds ratio [OR], 1.42; 95% confidence interval [CI], 1.22–1.66) indicated that males have a higher chance of survival after OHCA than females. However, the pooled estimate for adjusted effect estimates shows no difference in survival after OHCA between males and females (OR, 0.93; 95% CI, 0.84–1.03). Both meta‐analyses involved high statistical heterogeneity between studies: crude pooled estimate I^2^ = 95.7%, adjusted pooled estimate I^2^ = 91.3%. There does not appear to be a difference in survival between males and females when effect estimates are adjusted for possible confounding and/or mediating variables in non‐selected populations.

## 1 INTRODUCTION

### 1.1 Background

It is well‐known that males are more likely to experience out‐of‐hospital cardiac arrest (OHCA) than females.^1^, ^2^ For example, the age‐standardized incidence of OHCA in the Netherlands between 2009 and 2015 was 87.3 per 100,000 person‐years for males versus 30.9 per 100,000 person‐years for females.^3^ This difference is most likely due to males having a higher risk of cardiovascular disease (CVD) than females.^4^ However, in contrast to the well‐known sex differences in incidence of OHCA, the existing evidence on sex differences in survival after OHCA is inconclusive.^5^, ^6^, ^7^ For example, results from the ARREST study published in 2020 show that females have 40% lower odds of 30‐day survival after OHCA than males.^8^ In contrast, Kotini‐Shah et al^9^ reported that males have approximately 20% lower odds of 30‐day survival after OHCA than females. Some research suggests that intermediate variables, which lie on the causal pathway between sex and survival after OHCA, might explain this survival gap.^8^, ^10^ For example, among witnessed OHCAs, one study showed that females are less likely to be resuscitated than males (69.2% vs 73.9%, respectively),^8^ which subsequently decreases the chance of 30‐day survival after OHCA. Females are less likely to have an initial shockable rhythm than males (24% vs 42%, respectively).^8^, ^10^ Presenting with an initial shockable rhythm increases the chance of survival after OHCA dramatically.^10^

Three recent systematic reviews summarizing sex differences in survival after OHCA have reached contradictory conclusions, reporting lower survival in females,^11^ higher survival in females^12^ and no difference in survival.^13^ Feng et al^12^ included 11 studies that reported effect estimates adjusted for age, initial shockable rhythm, and witness status whereas Lei et al^11^ included studies reporting both crude and adjusted effect estimates, with 27 studies identified. The third review contained a selected subset of studies, largely only including those where patients had an initial shockable rhythm, received targeted temperature management (TTM), and had a witnessed arrest.^13^ Malik et al^13^ included 30 studies, all of which reported adjusted effect estimates. However, the heterogeneity of included study populations limits the interpretation of summary estimates from these analyses.

### 1.2 Importance

The differing inclusion criteria and heterogeneity in terms of pooling crude and/or adjusted effect estimates may account for the contradictory findings from these previous systematic reviews and meta‐analyses. However, it is important to examine why different conclusions have been reached to direct future research to focus on specific research areas and subsequently inform stakeholders to target these areas to improve any sex‐related survival gap after OHCA.

### 1.3 Goals of this investigation

Therefore, the aim of this systematic review and meta‐analysis is to synthesize the current evidence on sex differences in survival after OHCA in non‐selected populations only, with separate pooling of crude and adjusted effect estimates, to reconcile the conflicting conclusions from previous reviews on this topic.

## 2 METHODS

### 2.1 Study design and registration

This systematic review adheres to the Preferred Reporting Items for Systematic reviews and Meta‐Analyses (PRISMA) guidelines.^14^ The protocol of this review is registered with PROSPERO (CRD42021237887).

### 2.2 Search strategy

We systematically searched MEDLINE and Embase databases from inception to March 23, 2022 (Supporting Information Appendix SA data shows the entire search strategy). Reference lists of included studies were screened for potentially eligible articles.

### 2.3 Selection of studies

The records were loaded into Endnote citing software (v.X9; the Endnote Team; https://clarivate.com)^15^ and duplicates were removed. Title and abstract screening was done using Covidence systematic review software (v.2021; https://www.covidence.org).^16^ Potentially relevant articles were assessed for eligibility using the inclusion and exclusion criteria presented in Table 1. Only non‐selected population cohort studies were included in this systematic review. One reviewer (L.B.) screened all titles and abstracts yielded from the literature search, whereas a second reviewer (R.A.) assessed 10% of titles and abstracts retrieved from the search. This was done independently from the first reviewer (L.B.). Both reviewers (L.B. and R.A.) independently assessed all full texts of the retrieved articles after initial screening. Any differences in the assessment were discussed with a third reviewer (N.H.). Consensus had to be reached among the 3 reviewers in cases of difference in assessment. There were no instances of disagreement between reviewers.

**TABLE 1:** Inclusion and exclusion criteria for study selection.

	Inclusion	Exclusion
Study design	Observational studies (population‐based cohort studies)	Case reports Case control studies Cross‐sectional studies Systematic reviews RCTs
Participants	Adults (studies with a cohort containing both adult and pediatric patients were considered eligible) Non‐traumatic OHCA	Pediatric studies Myocardial infarction without cardiac arrest In‐hospital cardiac arrests Specifically looking at traumatic OHCA Specifically looking at COVID‐19 period
Exposure	Report on OHCA survival by sex	No reporting of OHCA survival in relation to sex Selected subgroups of patients with OHCA, for example, patients who received hypothermia, only patients with a shockable rhythm as initial rhythm or patients with OHCA caused by a specific illness
Outcome	Neurologically intact survival Survival until hospital discharge 30‐day survival	Survival until hospital admission Survival until ICU admission
Other	Any date of publication Any language	Results reported in conference abstracts only

Abbreviations: ICU, intensive care unit; OHCA, out‐of‐hospital cardiac arrest; RCT, randomized controlled trial.

### 2.4 Data extraction and synthesis

Two independent reviewers (L.B. and R.A.) extracted information on study characteristics, consisting of: number of patients, sex distribution figures, study design, characteristics of the study population, and effect estimates. Quality of studies and risk of bias was assessed independently by 2 reviewers (L.B. and R.A.) using the Newcastle‐Ottawa Scale (NOS).^17^ Disagreements between the independent reviewers were discussed with a third reviewer (N.H.). Consensus had to be reached among the 3 reviewers in cases of difference in assessment. There were no instances of disagreement between reviewers. Columns that were not relevant to any of the included studies (“Selection of the unexposed cohort,” “Outcome of interest not present at start of study,” “Follow‐up long enough for outcomes to occur,” “Adequacy of follow‐up of cohort”) were excluded from the NOS assessment. The outcome of interest in this systematic review is defined as survival to hospital discharge, 30‐day survival after OHCA or neurologically intact survival. Unfortunately, very few studies report on neurologically intact survival so therefore survival to hospital discharge and 30‐day survival was also included as a proxy for overall survival after OHCA. When studies reported multiple outcomes such as both neurologically intact survival and 30‐day survival only 1 was used, in the following order: neurologically intact survival, 30‐day survival, and survival until hospital discharge.

### 2.5 Data analysis

The primary analysis consisted of 2 separate meta‐analyses looking at the association between sex and survival after OHCA. The first included only crude effect estimates and the second included only adjusted effect estimates (adjusted for possible mediating and/or confounding variables). Notably, if studies reported both a crude effect estimate and an adjusted effect estimate they were included in both meta‐analyses.

Based on previous studies, considerable statistical between‐study heterogeneity was anticipated,^11^, ^12^ therefore a random‐effects model was used to pool effect estimates. The inverse‐variance method^18^ was used to pool pre‐calculated binary outcome effect sizes. Statistical heterogeneity was assessed using the Paule‐Mandel estimator^19^ to calculate the heterogeneity variance τ2 and the Higgins and Thompson I^2^ statistic.^20^ The Paule‐Mandel estimator is a recommended estimation method for univariate meta‐analysis, the τ2 statistic measures between‐study variance.^21^ The I^2^ statistic measures whether variation is likely due to chance or more likely due to study heterogeneity,^20^ I^2^ 0%–25%, I^2^ 26%–50%, and I^2^ ≥50% are respectively considered low, moderate, and high heterogeneity in this review.^22^

Forest plots were created to visualize the results of the meta‐analyses. A prediction interval is reported in the forest plots, this is an estimate of the between‐study variance in the random‐effects meta‐analyses.^23^ Subgroup analyses were planned a priori to address possible clinical heterogeneity^24^ and performed including only studies that adjusted for initial cardiac rhythm and/or bystander cardiopulmonary resuscitation (bCPR) (these are important mediating/confounding variables^10^, ^25^, ^26^).

To explore possible sources of high statistical heterogeneity, sensitivity analyses were performed.^27^, ^28^, ^29^, ^30^, ^31^ For the sensitivity analyses Baujat plots and forest plots were created (using the “leave‐one‐out method”) to assess that single studies have a disproportionately large effect on either heterogeneity or the pooled effect size. In further sensitivity analyses basic outliers, determined by statistical analysis, were removed. Publication bias was assessed using different methods, namely the Rücker's Limit Meta‐Analysis Method,^29^ the Duval and Tweedie Trim and Fill Method,^30^ and the Test for Right‐Skewness.^31^

All analyses were performed using R Statistical Software (v.4.0.3; R Core Team 2022; http://www.r‐project.org).^32^

## 3 RESULTS

**FIGURE 1:**
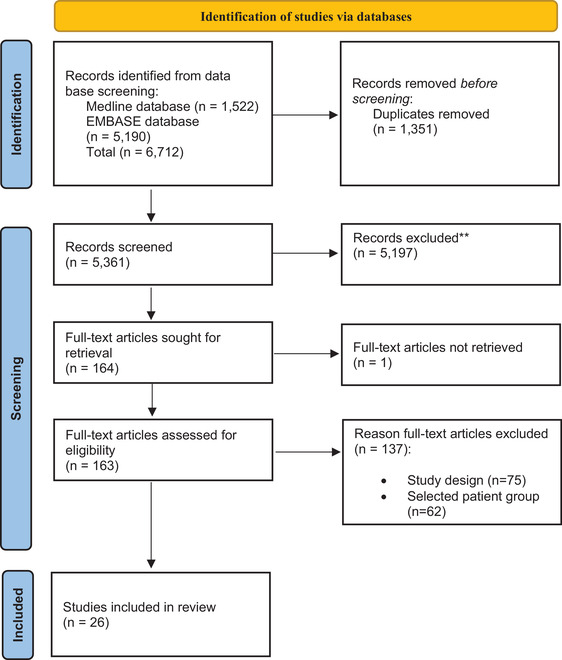
PRISMA flowchart study selection. *From*: Page MJ, McKenzie JE, Bossuyt PM, Boutron I, Hoffmann TC, Mulrow CD, et al. The PRISMA 2020 statement: an updated guideline for reporting systematic reviews. BMJ. 2021;372:n71. https://doi.org/10.1136/bmj.n71.

The initial search yielded 6712 articles and from these, 1351 duplicates were removed. After screening the studies by abstract and title, 164 studies remained. After screening full texts, 26 studies were eligible for inclusion in this systematic review (Figure 1 shows the selection process). Studies were mostly excluded based on having a selected population‐based setting.

### 3.1 Description of included studies

Baseline characteristics of included studies are summarized in Table 2. All included studies were cohort studies.^2^, ^7^, ^8^, ^9^, ^10^, ^33^, ^34^, ^35^, ^36^, ^37^, ^38^, ^39^, ^40^, ^41^, ^42^, ^43^, ^44^, ^45^, ^46^, ^47^, ^48^, ^49^, ^50^, ^51^, ^52^, ^53^ Twelve studies reported data from North‐America,^9^, ^10^, ^35^, ^36^, ^38^, ^43^, ^44^, ^45^, ^46^, ^47^, ^49^, ^52^ 7 from Europe,^8^, ^40^, ^41^, ^44^, ^48^, ^51^, ^53^ 6 from Asia,^2^, ^33^, ^34^, ^39^, ^42^, ^50^ and 2 from Oceania.^7^, ^37^ Five studies included pediatric patients as well as adult patients.^10^, ^43^, ^47^, ^48^, ^50^ Study size ranged from n = 477 to n = 386,535 and the proportion of female patients ranged from 18.6% to 45.1%. Eleven studies only included non‐traumatic OHCAs/OHCAs of presumed cardiac origin,^2^, ^7^, ^8^, ^10^, ^33^, ^36^, ^38^, ^42^, ^43^, ^44^, ^51^ the other 15 included OHCAs of all etiologies.^9^, ^34^, ^35^, ^37^, ^39^, ^40^, ^41^, ^45^, ^46^, ^47^, ^48^, ^49^, ^50^, ^52^, ^53^ This difference in etiology is unlikely to have had a major impact on the effect estimates as OHCAs of non‐cardiac origin account for a relatively small proportion of OHCAs.^54^ Of the 26 included studies, 3 studies only report crude estimates^8^, ^10^, ^41^ and of the other 23 studies, 7 studies report both crude and adjusted estimates^2^, ^9^, ^34^, ^37^, ^42^, ^47^, ^52^ and 16 only reported adjusted estimates.^7^, ^33^, ^35^, ^36^, ^38^, ^39^, ^40^, ^43^, ^44^, ^45^, ^46^, ^48^, ^49^, ^50^, ^51^, ^53^ Two studies adjusted only for age^34^, ^43^ and 2 studies did not state the variables adjusted for in the article.^42^, ^48^ Therefore, there is a large variation in variables adjusted for between the different studies. This could potentially be a major source of heterogeneity. Table 3 shows the results of the NOS^17^ risk of bias assessment of the included studies. The maximum number of awarded points was 5. The awarded points ranged from 3 to 5, indicating that studies were of moderate to high quality.

**TABLE 2:** Baseline characteristics of included studies.

Study	Country	Study period	Study population	Study size	% Male/female	Mean age, years (± SD)^a^	Study design	Adjusted for confounders/mediators
Ahn et al., 2012^33^	South Korea	2008	Data from nationwide, OHCA database; patients >20 years with symptoms of presumed cardiac etiology	N = 13,922 (male, 8764; female, 5158)	63.0/37.0	Male = 63.7 (14.8) Female = 72.4 (15.4)	Retrospective cohort	Age, location of arrest, witness status, response time, and transport time
Akahane et al., 2011^34^	Japan	2005–2007	Data from nationwide, OHCA database; patients 20–89 years old, all etiologies	N = 276,590 (male, 171,970; female, 104,620)	62.2/37.8	Male = 67.9 (15.2) Female = 72.7 (14.9)	Retrospective cohort	Age
Blewer et al., 2018^35^	United States	2011–2015	Data from Resuscitation Outcomes Consortium Registry; adults, non‐traumatic etiology	N = 19,331 (male, 12,225; female, 7106)	63.2/36.8	64.0 (17.0)	Retrospective cohort	Site, age, race, and bCPR
Blom et al., 2019^8^	The Netherlands	2006–2012	Data from ARREST registry; patients ≥20 years old, presumed cardiac etiology	N = 5717 (male, 4117; female, 1600)	72/28.0	Male = 66.2 (13.5) Female = 69.4 (14.6)	Retrospective cohort	
Bosson et al., 2016^36^	United States	2011–2014	Registry data from the Los Angeles County EMS system; adults, presumed cardiac etiology	N = 5174 (male, 3080; female, 2094)	59.5/40.5	Male = 66 (55–78) Female = 71 (59–82)	Retrospective cohort	Age, sex, initial rhythm, witness status, and bCPR
Bray et al., 2013^7^	Australia	2003–2010	Data from the VACAR; adults, presumed cardiac etiology	N = 10,453 (male, 7345; female, 3108)	70.3/29.7	Male = 69 (22) Female = 74 (19)	Retrospective cohort	Age, witnessed arrest, bCPR, year of arrest, rural location, public location, and EMS response time
Dicker et al., 2018^37^	New Zealand	2013–2015	Data from SOCAR; patients ≥16 years old, all etiologies	N = 3862 (male, 2678; female, 1184)	69.3/30.7	Male = 65 (53–76) Female = 68 (54–78)	Retrospective cohort	Age, location, etiology, initial rhythm, and witnessed status
Fairbanks et al., 2007^38^	United States	1998–2011	Cases were identified using a state‐mandated, EMS agency‐maintained list of all OHCA cases; adults, presumed cardiac etiology	N = 1177 (male, 958; female, 219)	81.4/18.6	Age = 67 (18–98)	Retrospective cohort	Age, sex, race, witnessed arrest, bCPR, initial rhythm and call‐response interval of 9 min or less
Goto et al., 2019^39^	Japan	2013–2016	Data from OHCA registry on the FDMA database server; adults, all etiologies	N = 386,535 (male, 217,173; female, 169,362)	56.2/43.8	Male = 74.3 (14.1) Female = 80.3 (13.2)	Retrospective cohort	Calendar year, rural/urban area, age, sex, witnessed arrest, initial rhythm, etiology, and bCPR
Herlitz et al., 2004^40^	Sweden	1990–2000	Data from the Swedish Cardiac Arrest Registry; adults, all etiologies	N = 24,917 (male, 17,149; female, 6648)	73.3/26.7	Male = 67 (16) Female = 69 (18)	Retrospective cohort	Age, witnessed arrest, bCPR, place of arrest, initial rhythm and etiology
Hubert et al., 2021^41^	France	2011–2017	Data from the RéAC; adults, all etiologies	N = 66,395 (male, 43,655; female, 22,740)	65.8/34.2	Male = 67 (57–78) Female = 74 (60–84)	Retrospective cohort	
Irfan et al., 2016^42^	Qatar	2012–2013	Data from the Qatar National OHCA registry; adults with presumed cardiac etiology	N = 447 (male, 360; female, 87)	80.5/19.5	Age = 51 (39–66)	Retrospective cohort	Not reported
Johnson et al., 2013^43^	United States	2005–2009	Data from the CARES data set; adults with presumed cardiac etiology	N = 19,398 (male, 11,745; female, 7653)	60.5/39.5	Male = 63 (52–75) Female = 69 (56–82)	Retrospective cohort	Age
Karlsson et al., 2015^44^	11 countries in Europe and the United States	2006–2012	Data from INTCAR; patients ≥12 years old, presumed cardiac etiology	N = 1667 (male, 1195; female, 472)	71.7/28.3	Male = 63 (53–72) Female = 62 (51–72)	Retrospective cohort	Sex, age, witnessed arrest, bCPR, time to ROSC, and initial shockable rhythm
Kim et al., 2001^45^	United States	1990–1998	Data from prehospital emergency medical system, Medic I; patients ≥30 years old, non‐traumatic OHCA	N = 10,879 (male, 7069; female, 3810)	65.0/35.0	Male = 66 Female = 71	Retrospective cohort	Initial shockable rhythm, age, witnessed arrest, bCPR, response time paramedic, and location of arrest
Kotini‐Shah et al., 2021^9^	United States	2013–2019	Data from the CARES data set; adults, non‐traumatic OHCA	N = 326,138 (male, 208,857; female, 117,281)	64.0/36.0	Male = 62 (22) Female = 65 (25)	Retrospective cohort	Demographics, cardiac arrest characteristics and bystander interventions; not specified further
May et al., 2018^46^	United States	2014–2016	Data from the CARES data set; adults, non‐traumatic OHCA	N = 2359 (male, 1296; female, 1063)	54.9/45.1	Age = 60.4 (20.5)	Retrospective cohort	Year of arrest, age, sex, location of arrest, witnessed arrest, and initial shockable rhythm
Morrison et al., 2016^47^	United States	2005–2007	Data from the ROC Epistry‐Cardiac Arrest Registry; pediatric and adult patients, non‐traumatic	N = 14,690 (male, 9350; female, 5340)	63.6/36.4	Male = 66 (25) Female = 73 (24)	Retrospective cohort	All Utstein predictors
Ng et al., 2016^2^	Seven countries in Pan‐Asia	2009–2012	Data from the PAROS Registry; adults, presumed cardiac etiology	N = 40,159 (male, 24,267; female, 15,892)	60.4/39.6	Male = 72 (60–81) Female = 82 (72–88)	Retrospective cohort	Age, sex, location, witnessed arrest, bCPR, initial rhythm, and response time
Pell et al., 2000^48^	Scotland	1988–1997	Data from the Heartstart (Scotland) database; pediatric and adult patients, all etiologies	N = 22,161 (male, 15,437; female, 6724)	69.7/30.3	Male = 65 (55–72) Female = 69 (59–76)	Retrospective cohort	Not reported
Rob et al., 2022^53^	Czech Republic	2012–2020	Data from the Prague OHCA Registry; adults, all etiologies	N = 960 (male, 693; female, 239)	75.1/24.9	Male = 60 (50–68) Female = 64 (54–75)	Retrospective cohort	Important resuscitation characteristics such as age, bCPR, initial shockable rhythm, and witnessed arrest; not specified further
Safdar et al., 2014^10^	Canada	1994–2002	Data from the OPALS database; ≥16 years old, presumed cardiac etiology	N = 10,862 (male, 7748; female, 3731)	65.7/34.3	Male = 69 (59–77) Female = 74 (64–82)	Retrospective cohort	
Teodorescu et al., 2012^49^	United States	2002–2007	Data from the Oregon‐SUDS; adults, all etiologies	N = 1296 (male, 865; female, 431)	66.7/33.3	Male = 63.2 (15.3) Female = 68.1 (15.8)	Retrospective cohort	Age, locations, disease burden, and SES
Wang et al., 2015^50^	Taiwan	2000–2012	Data from the NHIA and NHRI of Taiwan; pediatric and adult patients, all etiologies	N = 117,787 (male, NR; female, NR)	NR	NR	Retrospective cohort	Age, critical clinical conditions and features of health care organizations; not specified further
Winther‐Jensen et al., 2018^51^	Denmark	2007–2011	Data from a local Utstein‐based OHCA database; adults, presumed cardiac etiology	N = 704 (male, 529; female, 175)	75.1/24.9	Male = 64 (54‐73) Female = 71 (60–81)	Retrospective cohort	Age, comorbidity, and initial rhythm
Yamaguchi et al., 2017^52^	United States	1998–2013	Data from the ROC Epidemiological Registry (Epistry); adults, non‐traumatic	N = 2528 (male, 1663; female, 865)	65.8/34.2	NR	Retrospective cohort	Age, sex, location, witnessed arrest, initial rhyth, response time, bCPR, and non‐EMS AED shock

Abbreviations: AED, automated external defibrillator; ARREST, Amsterdam Resuscitation Studies; bCPR, bystander; CARES, Cardiac Arrest Registry to Enhanced Survival; CPR; CI, confidence interval; CPR, cardiopulmonary resuscitation; EMS, emergency medical services; INTCAR, International Cardiac Arrest Registry; IQR, interquartile range; NHIA, National Health Insurance Administration; NHRI, National Health Research Institutes; NR, not reported; OPALS, Ontario Prehospital Advanced Life Support; OR, odds ratio; Oregon‐SUDS, Oregon Sudden Unexpected Death Study; PAROS, Pan‐Asian Resuscitation Outcomes Study; RéAC, French National Cardiac Arrest Registry; ROC, Resuscitation Outcomes Consortium; ROSC, return of spontaneous circulation; SD, standard deviation; SES, socioeconomic status; SOCAR, St. John New Zealand OHCA Registry; VACAR, Victorian Ambulance Cardiac Arrest Registry.

^a^ Median (IQR) might be reported.

**TABLE 3:** Newcastle‐Ottawa Scale rating for included studies.

Study	Representativeness of the exposed cohort (1/5)	Ascertainment of exposure (1/5)	Control for important variable or additional variable (2/5)	Outcome assessment (1/5)	Total quality score (out of 5)
Ahn et al., 2012^33^	**+**	**+**	**+**	**+**	4
Akahane et al., 2011^34^	**+**	**+**	**+**	**+**	4
Blewer et al., 2018^35^	**+**	**+**	**+**	**+**	4
Blom et al., 2019^8^	**+**	**+**	**–**	**+**	3
Bosson et al., 2016^36^	**+**	**+**	**++**	**+**	5
Bray et al., 2013^7^	**+**	**+**	**+**	**+**	4
Dicker et al., 2018^37^	**+**	**+**	**++**	**+**	5
Fairbanks et al., 2007^38^	**+**	**+**	**++**	**+**	5
Goto et al., 2019^39^	**+**	**+**	**++**	**+**	5
Herlitz et al., 2004^40^	**+**	**+**	**++**	**+**	5
Hubert et al., 2021^41^	**+**	**+**	**–**	**+**	3
Irfan et al., 2016^42^	**+**	**+**	**+**	**+**	4
Johnson et al., 2013^43^	**+**	**+**	**+**	**+**	4
Karlsson et al., 2015^44^	**+**	**+**	**++**	**+**	5
Kim et al., 2001^45^	**+**	**+**	**++**	**+**	5
Kotini‐Shah et al., 2021^9^	**+**	**+**	**++**	**+**	5
May et al., 2018^46^	**+**	**+**	**++**	**+**	5
Morrison et al., 2016^47^	**+**	**+**	**++**	**+**	5
Ng et al., 2016^2^	**+**	**+**	**++**	**+**	5
Pell et al., 2000^48^	**+**	**+**	**+**	**+**	4
Rob et al., 2022^53^	**+**	**+**	**++**	**+**	5
Safdar et al., 2014^10^	**+**	**+**	**–**	**+**	3
Teodorescu et al., 2012^49^	**+**	**+**	**+**	**+**	4
Wang et al., 2015^50^	**+**	**+**	**+**	**+**	4
Winther‐Jensen et al., 2018^51^	**+**	**+**	**++**	**+**	5
Yamaguchi et al., 2017^52^	**+**	**+**	**++**	**+**	5

### 3.2 Meta‐analyses

**FIGURE 2:**
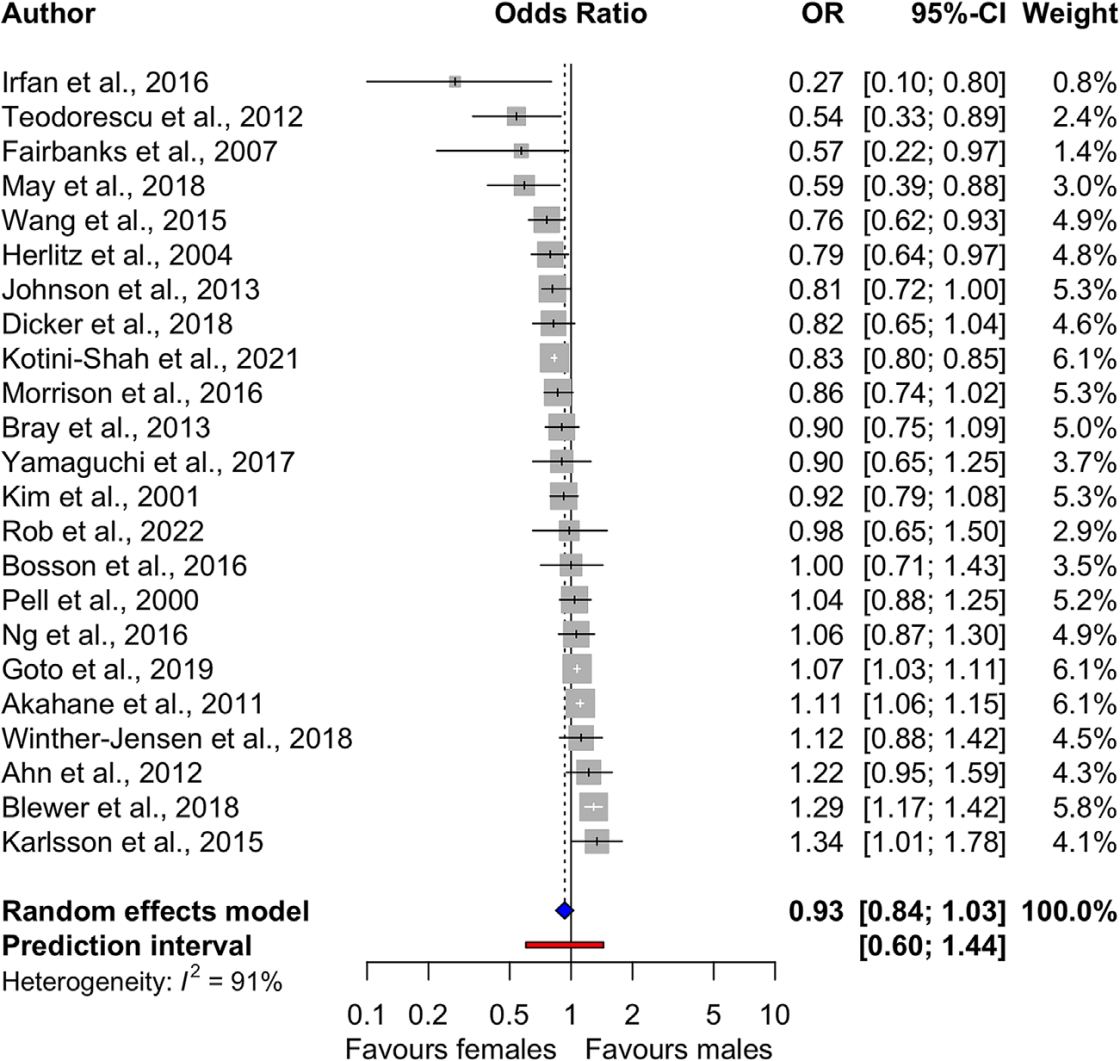
Forest plot meta‐analysis crude odds ratios.

**FIGURE 3:**
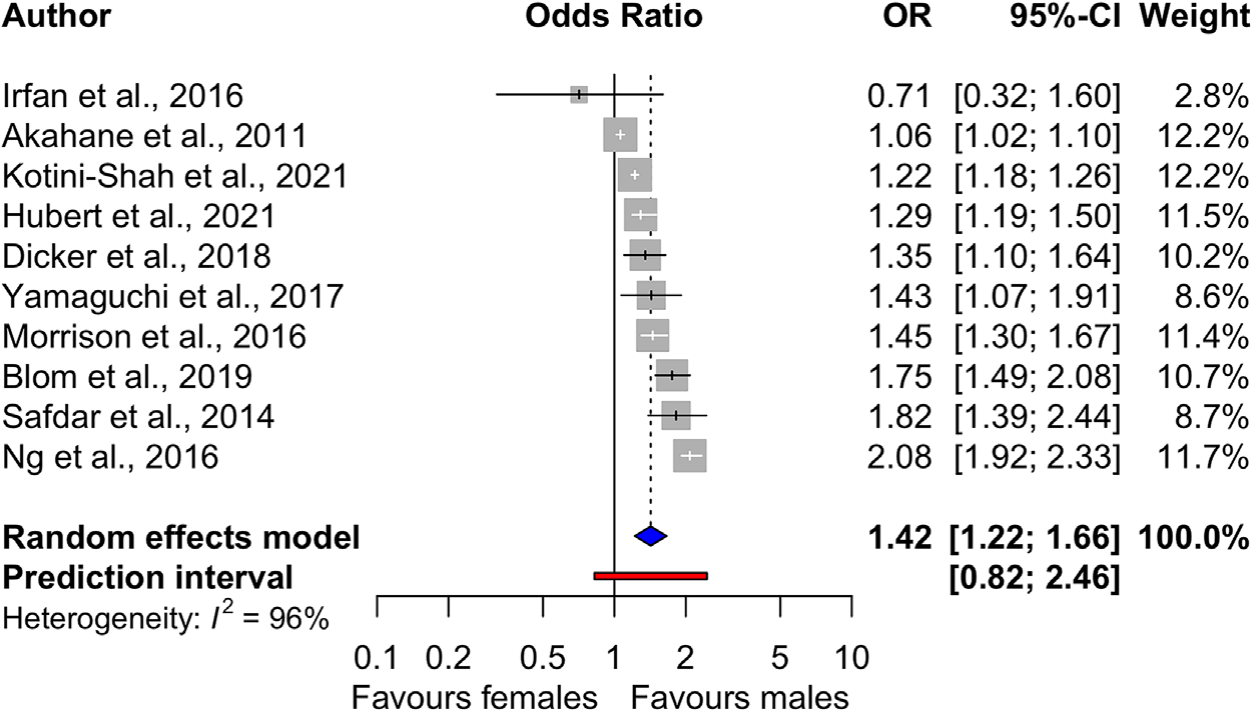
Forest plot meta‐analysis adjusted odds ratios.

The pooled analysis of 10 studies reporting crude odds ratios (ORs) for the association between sex and survival showed that male sex was significantly associated with increased odds of 30‐day survival after OHCA (OR, 1.42; 95% confidence interval (CI), 1.22–1.66) (Figure 2; Table 4). However, there was high statistical heterogeneity between studies (I^2^ = 95.7%; 95% CI, 93.7%−97.0%) and τ^2^ = 0.05). Pooled analysis of 23 studies reporting adjusted ORs for the association between sex and survival showed that sex was not associated with 30‐day survival after OHCA (OR, 0.93; 95% CI, 0.84–1.03) (Figure 3; Table 4). Again, there was high statistical heterogeneity between studies (I^2^ = 91.3%; 95% CI, 88.2%−93.5% and τ^2^ = 0.04).

**TABLE 4:** Results of meta‐analyses of crude‐ and adjusted effect estimates for the association between sex and OHCA survival.

	OR	95% CI	*P* value	I^2^ (%)	No. studies	No. patients
Crude effect estimates						
Main analysis	1.42	1.22–1.66	<0.001	95.7	10	747,388
Basic outliers removed^a^	1.24	1.18–1.31	<0.001	12.1	5	399,370
Influential cases removed^b^	1.41	1.24–1.60	<0.001	79.0	8	430,639
Adjusted effect estimates						
Main analysis	0.93	0.84–1.03	0.16	91.3	23	1,303,134
Basic outliers removed^c^	0.93	0.86–1.00	0.05	31.3	14	170,984
Influential cases removed^d^	0.91	0.82–1.00	0.05	55.3	19	280,618
Subset studies adjusted for initial rhythm^e^	0.93	0.84–1.02	0.13	89.9	12	494,651
Subset studies adjusted for witnessed^f^	0.94	0.86–1.02	0.15	88.7	14	540,483

Abbreviations: OHCA, out‐of‐hospital cardiac arrest; OR, odds ratio.

^a^ Removed as outliers: Akahane et al. (2011)^34^; Blom et al. (2019)^8^; Morrison et al. (2016)^47^; Ng et al. (2016)^2^; Safdar et al. (2014).^10^

^b^ Removed as influential cases: Akahane et al. (2011)^34^; Ng et al. (2016).^2^

^c^ Removed as outliers: Akahane et al. (2011)^34^; Blewer et al. (2018)^35^; Goto et al. (2019)^39^; Irfan et al. (2016)^42^; Karlsson et al. (2015)^44^; Kotini‐Shah et al. (2021)^9^; May et al. (2018)^46^; Teodorescu et al. (2012)^49^; Wang et al. (2015).^50^

^d^ Removed as influential cases: Akahane et al. (2011)^34^; Blewer et al. (2018)^35^; Goto et al. (2019)^39^; Kotini‐Shah et al. (2021).^9^

^e^ Removed for subset initial rhythm: Ahn^33^; Akahane^34^; Blewer^35^; Bray^7^; Irfan^42^; Johnson^43^; Kotini‐Shah^9^; Pell^48^; Rob^53^; Teodorescu^49^; Wang.^50^

^f^ Removed for subset witnessed: Akahane^34^; Blewer^35^; Irfan^42^; Johnson^43^; Kotini‐Shah^9^; Rob^53^; Teodorescu^49^; Wang^50^; Winther‐Jensen.^51^

### 3.3 Sensitivity and subgroup analyses

The pooled effect estimates did not change significantly when conducting the “leave‐one‐out method.” Heterogeneity was somewhat lower but still moderate to high (meta‐analysis crude effect estimates, I^2^ = 79.0%; meta‐analysis adjusted effect estimates, I^2^ = 55.3%). The results of the sensitivity and subgroup analyses were also in line with the results of the main meta‐analyses (Table 4). Results of the publication bias analyses indicate that publication bias is unlikely to have had a major impact on the results of the meta‐analyses (Table 5).

**TABLE 5:** Results of publication bias analyses.

	Crude effect estimates	Adjusted effect estimates
Rücker's limit method: analysis method crude	OR, 1.42 (95% CI, 1.22–1.66); *P* < 0.001	OR, 0.93 (95% CI, 0.84–1.03); *P* = 0.16
Rücker's limit method: analysis method adjusted	OR, 1.49 (95% CI, 1.25–1.77); *P* < 0.001	OR, 1.04 (95% CI, 0.92–1.17); *P* = 0.57
Duval and Tweedie trim‐and‐fill method	Added 3 studies OR, 1.20 (95% CI, 0.98–1.47); *P* = 0.07	Added 4 studies OR, 0.97 (95% CI, 0.86–1.10); *P* = 0.69
Test for right‐skewness	*P* χ^2^ value < 0.001; *P* values are right‐skewed	*P* χ^2^ value = 0.007; *P* values are right‐skewed

Abbreviations: CI, confidence interval; OR, odds ratio.

### 3.4 Limitations

The high statistical heterogeneity between studies included in the 2 meta‐analyses is a limitation of this systematic review. High statistical heterogeneity in systematic reviews is a common issue, especially when using observational studies in a meta‐analysis.^55^, ^56^ This finding is in line with other systematic reviews reporting on sex and survival after OHCA.^11^, ^12^, ^13^ The 3 systematic reviews recently published showed moderate (Feng et al (2021) I^2^ = 52.3%)^12^ to high (Lei et al (2020) I^2^ = 98.4%^11^ and Malik et al (2022) I^2^ = 89.1%)^13^ heterogeneity. The high heterogeneity is not unexpected as studies looking at the relationship between sex and survival after OHCA naturally vary in terms of included population, data collection and reporting, and adjustments made for confounding and/or mediating variables. Despite conducting several statistical methods to explore possible sources of heterogeneity, it is important to underline the high heterogeneity in the 2 meta‐analyses in this systematic review. Other important possible sources of high heterogeneity include the etiology of the OHCA (the initial cause of the cardiac arrest) and the specific inclusion and exclusion criteria of the cohorts used in the included studies as well as country‐specific EMS responses to OHCA. However, we consider all included articles clinically measuring roughly the same thing. Despite differences in approach of recognition and treatment of OHCA between countries, we consider the populations included in this systematic review to be similar enough to do meta‐analyses, despite the high heterogeneity.

Although we found no significant differences in pooled effect sizes between subgroups (meta‐analysis only including studies that adjusted for initial cardiac rhythm and meta‐analysis only including studies that adjusted for bCPR) in this review, it is possible that the subgroup analyses might not have had enough statistical power to highlight small differences.

Eligible articles might have been missed during the literature search if the articles did not mention sex or gender in either the abstract or the title. Ten percent of titles and abstracts were screened by the second reviewer (R.A.), this consisted of the review of 643 titles and abstracts and might be seen as a limitation. However, we felt 643 titles and abstracts and all full texts were enough to rule out any incidental mistakes. Another potential limitation of the search strategy is the wide time interval for study inclusions used. Resuscitation strategies have changed dramatically over time, and it is possible some strategies have favored one sex over the other in the past. One could also argue that looking at neurologically intact survival is more important than survival to hospital discharge or 30‐day survival. Unfortunately, reporting on neurologically intact survival seems more complicated than reporting on survival to hospital discharge or 30‐day survival and therefore very few studies report on neurologically intact survival.

## 4 DISCUSSION

This systematic review included 26 cohort studies undertaken in non‐selected populations, reporting on the association between sex and short‐term OHCA survival. Pooled estimates based on crude effect estimates suggest a sex difference in 30‐day survival after OHCA favoring males. Pooled estimates based on adjusted effect estimates do not suggest any sex difference in 30‐day survival after OHCA.

Important sex‐based differences in cardiac arrest characteristics that influence survival after OHCA exist. For example, the incidence of small vessel disease is higher in females than in males,^57^, ^58^ whereas the incidence of myocardial infarction is higher in males than in females.^59^, ^60^ These differences are important as the etiology of OHCA influences survival after OHCA.^61^, ^62^, ^63^ Furthermore, females are less likely to present with an initial shockable rhythm^8^, ^53^ and less likely to receive bCPR than males.^8^, ^64^ The difference in initial shockable rhythm might partially be explained by the fact that females delay calling emergency medical services (EMS) when experiencing symptoms of acute myocardial infarction because the symptoms differ between males and females, although the magnitude of this impact is unknown.^65^, ^66^ On average females are likely to be older than males when experiencing OHCA.^67^ All these differences have been reproduced and reported multiple times by large studies like OPALS^10^ and CARES.^9^ Beyond the prehospital resuscitation phase, differences have been shown in the way sex neutral protocols for the management of acute coronary syndromes are applied to males versus females, with males more likely to receive treatment.^68^, ^69^ Some of these variables might explain the male survival benefit found in crude effect estimates. When looking at the effect estimates adjusted for these characteristics, the male survival benefit seems to have disappeared and therefore it seems likely that the crude survival difference between the sexes is due to differences in OHCA characteristics. Most of these variables are considered to be mediators and not confounders, therefore they should be handled differently than confounders and not be adjusted for when conducting regression analysis.

A key rationale for the current systematic review was to explore reasons for the conflicting results of 3 fairly recently published systematic literature reviews and provide clarity on this topic for research user groups.^11^, ^12^, ^13^ Our results indicate that the contrasting finding that survival is lower in females than males from Lei et al^11^ is due to the inclusion of both crude and adjusted effect estimates within a single meta‐analysis. Feng et al^12^ only included effect estimates that adjusted for age, witnessed status, and initial cardiac rhythm. This is sensible, given the likely effect of these variables on outcome.^8^, ^70^ In line with this, we performed subgroup analyses looking at initial cardiac rhythm and bCPR. When comparing Malik et al^13^ with our current study, it is clear that Malik et al^13^ included mainly studies that reported findings on specific, selected patients' groups of the OHCA population that are not representative of the total population of adult non‐traumatic OHCA patients. Notably, our findings underline the need for transparency in reporting details of analyses. To illustrate this, some articles reporting results of primary studies do not specify for which variables adjustments were made in the analyses.^42^, ^48^ The need to adjust for possible confounders and making the distinction between mediators and confounders in regression analyses is important.^71^ Crude effect estimates and effect estimates that are adjusted for possible confounding and/or mediating variables can yield very different results and subsequent conclusions.^11^, ^12^

The search strategy used in this systematic review was comprehensive and used multiple databases to identify current available literature on sex differences in survival after OHCA. There are 9 studies^9^, ^35^, ^38^, ^41^, ^42^, ^44^, ^50^, ^52^, ^53^ included in this systematic review that were not included in either Feng et al,^12^ Lei et al,^11^ or Malik et al.^13^ In this systematic review crude effect estimates and adjusted effect estimates are discussed separately as opposed to the study of Lei et al.^11^ This is a major strength because, as demonstrated here, it can have a large impact on the results of the meta‐analysis and has partly explained the conflicting results of the earlier published systematic reviews. This systematic review includes sensitivity analyses and subgroup analyses, which add to the validity and robustness of the results. According to Schwarzer et al,^72^ subgroup sensitivity analyses are only sensible when the meta‐analysis includes at least 10 studies, as was the case in our review. Another strength of this systematic review is that the included studies are all based on non‐selected populations, in contrast with other systematic reviews that included selected subgroups within the OHCA population.^11^, ^13^ For example, a subgroup of patients who have undergone TTM have at least survived until hospital admission, therefore that group is not representative of the whole OHCA population and can distort results because of the introduction of selection bias. In contrast with other systematic reviews,^11^, ^13^ our review only included studies reporting on survival until hospital discharge, 30‐day survival after OHCA or neurologically intact survival after OHCA. Studies reporting survival until hospital admission were excluded because a substantial number of people will be pronounced dead either in the emergency department or at the intensive care unit.^73^, ^74^ Therefore, we consider survival until hospital admission to be a bad proxy for long‐term survival after OHCA.

Although the results of this systematic review might not have immediate implications for clinical practice, it might provide an explanation as to where the differences between crude and adjusted survival between the sexes are coming from. This is important, because it could direct future research to focus on which specific variables or resuscitation characteristics contribute the most to the differences between males and females in crude survival and handle these variables as mediators instead of confounders in regression analyses. Subsequently, policies and governmental strategies might focus on these characteristics in the future. For example, initiatives such as bCPR training using female mannequins to improve female bCPR rates might be useful to target the lower bCPR rates in females compared with males.

The meta‐analysis that includes effect estimates of analyses which have adjusted for variables that may confound or mediate the association between sex and 30‐day survival after OHCA suggests no association. However, when looking at crude effect estimates there are differences in male and female odds of 30‐day survival after OHCA as well as differences in rates of possible mediating variables such as bCPR, initial cardiac rhythm and other variables which have a substantial impact on survival after OHCA. Therefore, these variables might partially explain the lower chances of OHCA survival among females compared with males when looking at crude effect estimates. Further research is needed to establish which specific variables or resuscitation characteristics contribute the most to the differences in rates of 30‐day survival after OHCA between males and females.

## AUTHOR CONTRIBUTIONS

**Laura A. E. Bijman**: Conceptualization, methodology, formal analysis, data curation, writing–original draft, and visualization. **Raied Alotaibi**: Validation and writing–review and editing. **Caroline A. Jackson**: Methodology and writing–review and editing. **Gareth Clegg**: Writing–review and editing. **Nynke Halbesma**: Conceptualization, methodology, writing–review and editing, and supervision.

## CONFLICTS OF INTEREST STATEMENT

The authors declare no conflicts of interest.

## Supporting information

Supporting Information [SKQ]:AU: Please confirm that (1) this article contains Supporting Information, (2) that all the item(s) is/are cited in text and in the correct locations, and (3) all supporting materials have been provided.
