# Supplementary material for: Association between sex and survival after out‐of‐hospital cardiac arrest: A systematic review and meta‐analysis
Source: J Am Coll Emerg Physicians Open. 2023 Apr 29;4(3):e12943. doi: 10.1002/emp2.12943 (PMC10148381; doi:10.1002/emp2.12943)
Supplement: Supplementary file 1 — Supporting Information [SKQ]:AU: Please confirm that (1) this article contains Supporting Information, (2) that all the item(s) is/are cited in text and in the correct locations, and (3) all supporting materials have been provided. [file EMP2-4-e12943-s001.pdf]

Appendix A. Supplementary data
Supplement 1: complete search strategy

Ovid MEDLINE(R) and Epub Ahead of Print, In-Process & Other Non-Indexed Citations, Daily and Versions(R) 1946 to March 23, 2022

1. Out-of-Hospital Cardiac Arrest/
2. (Out of Hospital Cardiac Arrest? or OHCA?).ab,kf,ti.
3. 1 or 2
4. heart arrest/
5. (cardiac arrest? or heart arrest? or cardiopulmonary arrest? or scd or sudden cardiac arrest or sca).ab,kf,ti.
6. 4 or 5
7. resuscitation/ or emergency medical services/
8. (prehospital or pre hospital or ambulance? or preadmission or pre admission or CPR or automatic external defibrillator or AED or bystander? or cardiopulmonary resuscitation or resuscitat?).ab,kf,ti.
9. 7 or 8
10. 6 and 9
11. 3 or 10
12. gender/
13. sex/
14. (men or women or gender or sex or male or female).ab,kf,ti.
15. 12 or 13 or 14
16. mortality/ or survival rate/
17. (hospital discharge or surviv? or mortality or death or alive or dying or outcome).ab,kf,ti.
18. 16 or 17
19. 11 and 15 and 18
20. limit 19 to humans

Search results: 1,522

Embase Classic+Embase 1947 to March 23, 2022

1. Out-of-Hospital Cardiac Arrest/
2. (Out of Hospital Cardiac Arrest? or OHCA?).ab,kw,ti.
3. 1 or 2
4. heart arrest/
5. (cardiac arrest? or heart arrest? or cardiopulmonary arrest? or scd or sudden cardiac arrest or sca).ab,kw,ti.
6. 4 or 5
7. resuscitation/ or emergency medical services/
8. (prehospital or pre hospital or ambulance? or preadmission or pre admission or CPR or automatic external defibrillator or AED or bystander? or cardiopulmonary resuscitation or resuscitat?).ab,kw,ti.
9. 7 or 8
10. 6 and 9
11. 3 or 10
12. gender/
13. sex/
14. (men or women or gender or sex or male or female).ab,kw,ti.
15. 12 or 13 or 14
16. mortality/ or survival rate/
17. (hospital discharge or surviv? or mortality or death or alive or dying or outcome).ab,kw,ti.
18. 16 or 17
19. 11 and 15 and 18
20. limit 19 to humans

Search results: 5,190

<u>Total:</u> 6,712
